# Crabs *Eriocheir japonica* and *Paralithodes camtschaticus* Are a Rich Source of Lipid Molecular Species with High Nutritional Value

**DOI:** 10.3390/foods12183359

**Published:** 2023-09-07

**Authors:** Ekaterina V. Ermolenko, Tatyana V. Sikorskaya, Valeria P. Grigorchuk

**Affiliations:** 1 A.V. Zhirmunsky National Scientific Center of Marine Biology, Far Eastern Branch, Russian Academy of Sciences, ul. Palchevskogo 17, 690041 Vladivostok, Russia; miss.tatyanna@yandex.ru; 2 Federal Scientific Center of the East Asia Terrestrial Biodiversity, Far Eastern Branch, Russian Academy of Sciences, Pr-t 100-Letiya Vladivostoka 159, 690022 Vladivostok, Russia; kera1313@mail.ru

**Keywords:** crab, lipid, mass spectrometry, nutritional value

## Abstract

Due to their valuable meat and hepatopancreas, the world’s most famous delicacies, crabs, have become target species of commercial fisheries and aquaculture. By methods of supercritical fluid and high-performance liquid chromatography, coupled with high resolution mass spectrometry, we analyzed triacylglycerols (TG) and phospholipids (PL)—glycerophosphoethanolamines (PE), glycerophosphocholines (PC), glycerophosphoserines (PS), and glycerophosphoinositols (PI)—in the hepatopancreas and muscles of the Japanese mitten crab *Eriocheir japonica* and the red king crab *Paralithodes camtschaticus* inhabiting the Sea of Japan. TGs were the main class of lipids in the crab hepatopancreas, while they were found in trace amounts in muscle. TGs of *E. japonica* differed from those of *P. camtschaticus* by a higher content of 16:0, 16:1, 18:2, and 20:4 FA and a lower content of eicosapentaenoic and docosahexaenoic acids. The Japanese mitten crab differed from the red king crab by a lower content of molecular species with eicosapentaenoic acid in PC and PI; an increased content of arachidonic acid in PE, PS, and PI; and a lower content of molecular species with docosahexaenoic acid in PE in the hepatopancreas and muscles. The high nutritional value of the crabs *E. japonica* and *P. camtschaticus* was confirmed by a high content of molecular species of lipids with n-3 polyunsaturated fatty acids. The data of the lipid molecular species profile provide new background information for future studies on biochemistry and aquaculture of crabs.

## 1. Introduction

Crabs belong to a large group of invertebrates, of which some are valuable aquaculture and commercial fishery species. About one million tons of crabs are produced each year at aquaculture farms globally [1]. One of the most popular crab species farmed is the Chinese mitten crab (*Eriocheir sinensis*). King crab, of the genus Paralithodes, *P. camtschaticus*, is among the major commercial species from the northern Pacific Ocean [2]. The edible parts of crabs, being the world’s most famous delicacy, also have valuable nutritional properties. Crab meat contains essential amino acids, minerals, and vitamins [3,4]. Chitosan is obtained from the crab carapace, which has antibacterial, antioxidant, immune, and antitumor activities [5].

One of the important components of crabs’ nutritional value are lipids, including n-3 polyunsaturated fatty acids (PUFAs). The PUFA content of many crab species has been determined [2,6,7]. In utero and in the early years of a child’s life, n-3 PUFA significantly improves mental development and maturation of visual function [8]. The biological activity of n-3 PUFAs is mainly related to their anti-inflammatory properties [9], which have great implication in management of a number of neurodegenerative diseases [10] and breast cancer [11].

Currently, the lipidomic approach to biochemical studies of marine invertebrates is of particular importance [12]. These studies focus on the lipid molecular species profile, their biological activities, subcellular localization, and tissue distribution, as well as lipid metabolism and lipid-mediated signaling processes that regulate cellular homeostasis in health and disease [13,14]. The sensitivity of the lipidome to such factors as genotype, microbiota, and diet makes lipidomics the base to study interactions between genes, diet, nutrients, and metabolism [13]. Data on compositions of the crab lipid molecular species are increasingly accumulated. To date, the lipidomes of five crab species (*Paralithodes camtschaticus*, *Portunus trituberculatus*, *Eriocheir sinensis*, *Cancer magister*, and *Cancer pagurus*) have been studied for purpose of species discrimination [1]. The lipid molecular profiles of species were also assessed to elucidate the pathogenesis of protozoans in *E. sinensis* [15]; the metabolic response to various dietary n-3 PUFA in juvenile *P. trituberculatus* [16]; the embryonic development of two sympatric brachyuran crabs (*Carcinus maenas* and *Necora puber*) [17]; and the nutritional value of the crab *P. trituberculatus* depending on the diet during its cultivation [18].

One of the species for cultivation in the Sea of Japan is the Japanese mitten crab *Eriocheir japonica*, widely distributed in eastern Asia including the waters off Sakhalin, Primorsky Krai of Russia, eastern Korea, the Japanese archipelago (except the Ogasawara Islands), Taiwan, and Hong Kong [19]. *Eriocheir japonica* crab was previously shown to be rich in n-3 PUFA [7]. The red king crab *P. camtschaticus* rich n-3 PUFA is the commercial and expensive species, but the decline of natural populations requires the development and introduction of biotechnics for artificial reproduction of this species [2,20]. Our study aimed to compare the lipid molecular species compositions of the muscles and hepatopancreas of the edible crabs *E. japonica* and *P. camtschaticus* inhabiting the Sea of Japan. This study may extend our knowledge of the distribution of lipids in crab tissues and the nutritional value of crab lipids.

## 2. Materials and Methods

### 2.1. Sample Collection

A red king crab (a male weighing 2950 ± 140 g) was caught in November in the Sea of Japan and provided for research by the Group of Companies “Antey” (Vladivostok, Russia). A Japanese mitten crab (a male weighing 250 ± 40 g) was caught in October in the Sea of Japan by a diver of the A.V. Zhirmunsky National Scientific Center of Marine Biology. Four of each crab species were taken for lipid analysis. The hepatopancreases and muscles were manually collected from the crabs and then immediately subjected to lipid extraction.

### 2.2. The Lipid Analysis

PL standards, including phosphatidylcholine (12:0/12:0 PC), phosphatidylethano-lamine (18:0/18:1 PE,), phosphatidylserine (16:0/16:0 PS), phosphatidylinositol (18:0/18:0 PI), sphingomyelin (d18:1/16:0 SM), phosphatidic asid (16:0/16:0 PA), triacylglycerol (16:0/16:0/18:1 TG), diacylglycerol (16:0/16:0 DG), and cholesterol were purchased from Avanti Polar Lipids, Inc. (Alabaster, Al, USA). Isopropanol and *n*-hexane (MS grade) were purchased from Honeywell Riedel-de Haen (Charlotte, NC, USA). Ammonia solution, triethylamine, and formic acid were purchased from Sigma-Aldrich (St. Louis, MO, USA). The ultrapure water was obtained using a Milli-Q water purification system (Millipore, MA, USA). Chloroform and methanol (analytical grade) were purchased from Vekton (St. Petersburg, Russia).

Internal standards 12:0/12:0 PC and 16:0/16:0/18:1 TGs were added to each sample allowing for further quantitative analysis. Total lipids were extracted with a chloroform/methanol mixture according to [21], evaporated under reduced pressure, weighed, dissolved in chloroform and stored at −40 °C under argon. To analyze the content and structure of the molecular species of phosphorus-containing lipids, total lipids were separated on a Shim-Pack diol column (4.6 × 50 mm, particle size 5 μm) (Shimadzu, Japan) using a Nexera-e chromatography system (Shimadzu, Japan). The elution condition has been earlier described in detail in [22]. A high-resolution tandem mass spectrometer LCMS-IT-TOF (Shimadzu, Japan) was used in the identification and quantification of lipids. Analysis was performed under the electro-spray ionization (ESI) mode with simultaneous registration of signals of positive and negative ions. Scanning was performed in a *m*/*z* range of 100–1400. Source voltage was −3.5 kV in case of negative ion formation and 4.5 kV in case of positive ion formation. The temperature of the ion source was 200 °C; dry gas (N_2_) pressure, 150 kPa; the flow rate of nebulizing gas (N_2_), 1.5 L/min. Argon (0.003 Pa) was used in the collision chamber of the mass spectrometer. The structural identification of each lipid molecular species was conducted by comparing the retention times, ion forms, obtained ratio *m/z* within 5–7 ppm accuracy, and specific fragmentation behaviors of the phospholipid classes with the commercially available lipid standards. Detailed information on identification was described earlier in [22,23]. The percentages of the individual molecular species of each lipid class were calculated on the basis of peak area of negative ions [M–H]^−^, except for PC and SM which were estimated on the basis of peak area of negative ions [M+HCOOH]^−^ and positive ions [M+H]^+^, respectively. A correction coefficient used in the elimination of differences in the phospholipid’s ionizations. The coefficients were calculated as the ratio of the areas of individual PL standards to the area of the internal standard (PC 12:0/12:0). The peak areas of molecular species of analyzed crab lipids were calculated using the obtained coefficients.

The molecular species of neutral lipids (sterols (ST), DG, and TG) were separated by supercritical fluid chromatography on a Nexera UC system (Shimadzu, Japan) using two sequential Shim-pack XR-ODSII (2.0 × 150 mm) columns (Japan) under isocratic elution (0.6 mL min^–1^) with supercritical CO_2_ supplemented with 35% methanol. To measure the amounts of the molecular species of TG, DG and sterols, an ELSD LT II (Shimadzu, Japan) light scattering detector was used (the evaporating tube temperature was 40 °C; the spraying gas (N_2_) pressure was 0.4 MPa). The structures of the molecular species were determined on a high-resolution tandem mass spectrometer LCMS-IT-TOF (Shimadzu, Japan); HCOOH (0.1% in MeOH) was added to the eluent flowing out of the chromatographic column using a pump-through post column micromixer (0.2 mL min^–1^). The analysis was performed under atmospheric pressure with chemical ionization (APCI) in the positive ion mode. Spectra were recorded at *m/z* 150–1100. The interface temperature was 350 °C and the desolvation line temperature was 200 °C. The rates of nebulizing (N_2_), heating (air), and drying (N_2_) gas supplies were 3, 10, and 10 L/min, respectively. The identification of molecular species of TG was performed according to published previously study [22].

### 2.3. Statistical Analysis

The raw data obtained were tested for homogeneity of variances (Levene’s test) and normality of data distribution (Shapiro–Wilk’s test). Differences in the mean concentration of PL molecular species (% of each PL class) were analyzed by two-way analysis of variance (ANOVA). The factors were the origin (species) and the tissue (muscle and hepatopancreas). Both factors were fixed. The significant differences between the levels within the factors were assessed post hoc using Tukey’s HSD test. All statistical analyses were performed using the STATISTICA 12 package (StatSoft, Inc., Tulsa, OK, USA). A probability level of *p* < 0.05 was considered statistically significant. Values are presented as mean ± standard deviation. Heat maps were built using R statistical software (ver. 4.3.1, R Core Team).

## 3. Results

A high lipid content was recorded from the crab hepatopancreas. The Japanese mitten crab was distinguished by an increased lipid content of the hepatopancreas (109.63 ± 0.61 mg/g wet weight (w.w.) of tissue). The hepatopancreas of the red king crab contained lipids at 36.82 ± 3.64 mg/g w.w. The muscles were characterized by a low lipid content (3.53 ± 0.92 mg/g w.w. for *E. japonica* and 1.60 ± 0.40 mg/g w.w. for *P. camtschaticus*). The major lipid classes in the hepatopancreases of the studied crabs were TG, sterols, PC, and PE (Table 1). PC, PE, and diacylglycerols dominated crab muscles. Sphingomyelins (SM), phosphatidic acids (PA), and lysophosphatidylethanolamines (LPE) were also identified in the crab lipids.

MS^2^ fragmentation allows for characterization of the composition of lipid molecular species. In the muscles and hepatopancreas of *E. japonica* crab, 151 and 196 lipid molecular species were found, respectively (Table S1). The muscles and hepatopancreas of *P. camtschaticus* contained 152 and 178, respectively (Table S1).

**Figure 1:**
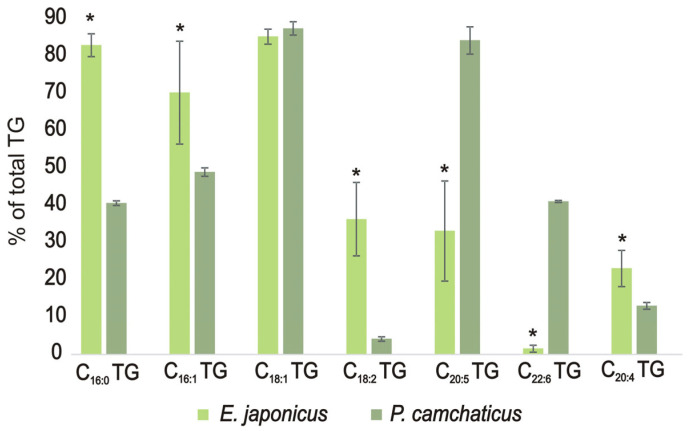
The content of TG molecular species contained the main fatty acids in the hepatopancreas of the crabs *Eriocheir japonica* and *Paralithodes camtschaticus.* Data are expressed as the mean ± SD, *n* = 4 (* *p* < 0.05). TG, triacylglycerols.

The TG compositions of the hepatopancreases from the studied crabs had significant differences (Tables S2 and S3). *E. japonica* crab contained 98 molecular species of TG; in *P. camtschaticus* lipids, 59 molecular species were detected. Only six TGs were similar between the hepatopancreases of both crabs. TGs were compared on the basis of the presence of major fatty acids, which are part of the molecular species (Figure 1). The *E. japonica* hepatopancreas contained mainly C_16:0_, C_16:1_, and C_18:1_ fatty acids (FAs). Among unsaturated FAs, C_18:2_, C_20:5_, and C_20:4_ FAs were present in *E. japonica* in considerable amounts. The major molecular types of TG in the Japanese mitten crab hepatopancreas were 20:4/16:0/18:1 TG (7.63 ± 1.28% of total TG) and 16:1/14:0/16:1 TG (9.82 ± 0.72% of total TG). The red king crab hepatopancreas was characterized by a high content of TG with C_18:1_ and C_20:5_ fatty acids, among which the major one was 20:5/18:1/18:1 TG (10.21 ± 0.50% of total TG).

**Figure 2:**
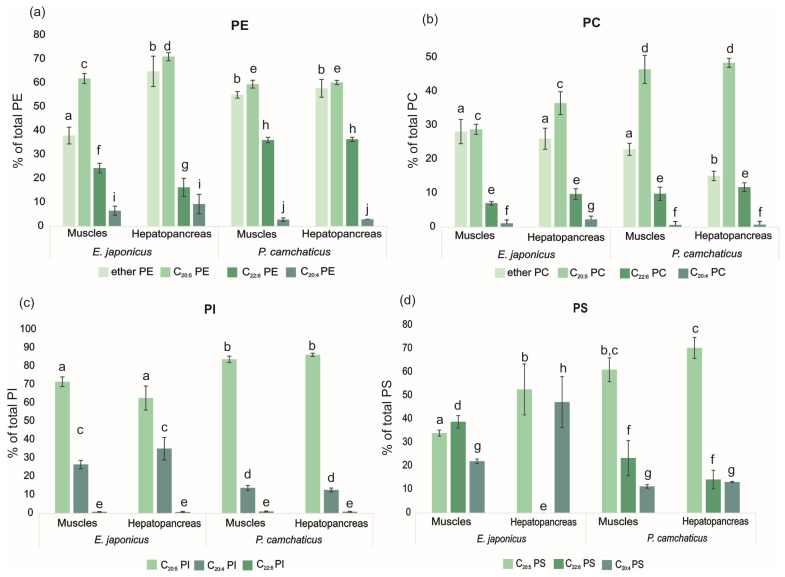
(**a**) The comparison of crab tissues by molecular species of phospholipids containing ether bond, C_20:5_, C_22:6_, and C_20:4_. (**b**) Data are expressed as the mean ± SD (*n* = 4). (**c**) Different letters indicate significant difference among different samples (HSD test, *p* < 0.05). (**d**) PE: glycerophosphoethanolamines; PC: glycerophosphocholines, PI: glycerophosphoinositols; PS: glycerophosphoserines.

The PE consisted primarily of eicosapentaenoic (C_20:5_, EPA) and docosahexaenoic acids (C_22:6_, DHA) in all the samples studied (Table S4). The alkyl fragment was present at the *sn*-1 position of glycerol (ether bond) in 38.09 ± 3.50% and 64.97 ± 3.38% of total PE in the muscle and hepatopancreas samples of *E. japonica*, respectively (Figure 2a). The *P. camtschaticus* contained alkyl/acyl PE at 55.13 ± 1.37% of total PE in muscles and 57.85 ± 3.73% of total PE in the hepatopancreas. The major PE molecular species with a more than 5% content of total PE in the studied samples were 18:1 alk/20:5 PE, 18:0 alk/20:5 PE, 18:0/20:5 PE, 18:1/20:5 PE, 18:1 alk/22:6 PE; 18:0 alk/22:6 PE, and 18:1/22:6 PE. The muscle and hepatopancreas samples of *E. japonica* were distinguished by a different level of alkylacyl PE: C_20:5_ PE and C_22:6_ PE (HSD test, *p* < 0.05) (Figure 2a). The lipids of the *P. camtschaticus* tissues showed a higher level of C_22:6_ PE (HSD test, *p* < 0.05) than in the *E. japonica* tissues. The *E. japonica* hepatopancreas contained a higher level of C_20:4_ PE (6.62 ± 1.90% of total PE) compared to the *P. camtschaticus* hepatopancreas (2.91 ± 0.68% of total PE).

The PC composition is provided in Supplementary Materials (Table S4). The major PC molecular species in the studied samples were 18:1/16:0 PC, 16:0/20:5 PC, 16:1/20:5 PC, 18:1/18:1 PC, 18:1/20:5 PC, and 18:0/20:5 PC (Table S4). No significant differences of PC groups between the tissue types were found in both crabs (Figure 2b). The *P. camtschaticus* hepatopancreas contained the lowest level of alkylacyl PC compared to *P. camtschaticus* muscles and *E. japonica* tissues (HSD test, *p* < 0.05). Lipids of the *P. camtschaticus* samples had a higher level of PC with EPA compared to the *E. japonica* crabs (HSD test, *p* < 0.05) (Figure 2b).

The PI composition was represented mainly by the following molecular species: 16:0/20:5 PI, 16:0/20:4; 18:0/20:5 PI, 18:1/20:5 PI, 18:0/20:4 PI, and 20:1/20:5 PI (Table S4). The hepatopancreas of the studied crabs differed from muscles by the presence of 18:2/20:5 PI and 20:5/20:5 PI. A comparison of the crab species showed that the *E. japonica* tissues differed from those of the *P. camtschaticus* samples by an elevated amount of PI with ARA and a reduced amount of PI with EPA (Figure 2c).

The major FA in PS were 18:0, 20:4, 20:5, and 22:6 (Table S4). The *E. japonica* hepatopancreas did not contain PS with DHA, whereas tissues of *P. camtschaticus* and muscles of *E. japonica* consisted of a higher amount of DHA-containing PS compared to other PL. Lipids of the *E. japonica* hepatopancreas included only two molecular species of PS (18:0/20:4 PS and 18:0/20:5 PS). The muscles of *P. camtschaticus* differed from the hepatopancreas by the presence of 20:1/22:6 PS. All the tissues of *E. japonica* contained a higher level of ARA in PS compared to those in the *P. camtschaticus* samples (HSD test, *p* < 0.05). The *E. japonica* muscles were characterized by a high level of PS molecular species with DHA (Figure 2d).

The crab phospholipid composition included SM, PA, and LPE (Table S5), but we did not consider these lipid classes in the comparison of crabs. We did not determine the molecular species composition of SM either. The LPE and PA were present in small amounts.

**Figure 3:**
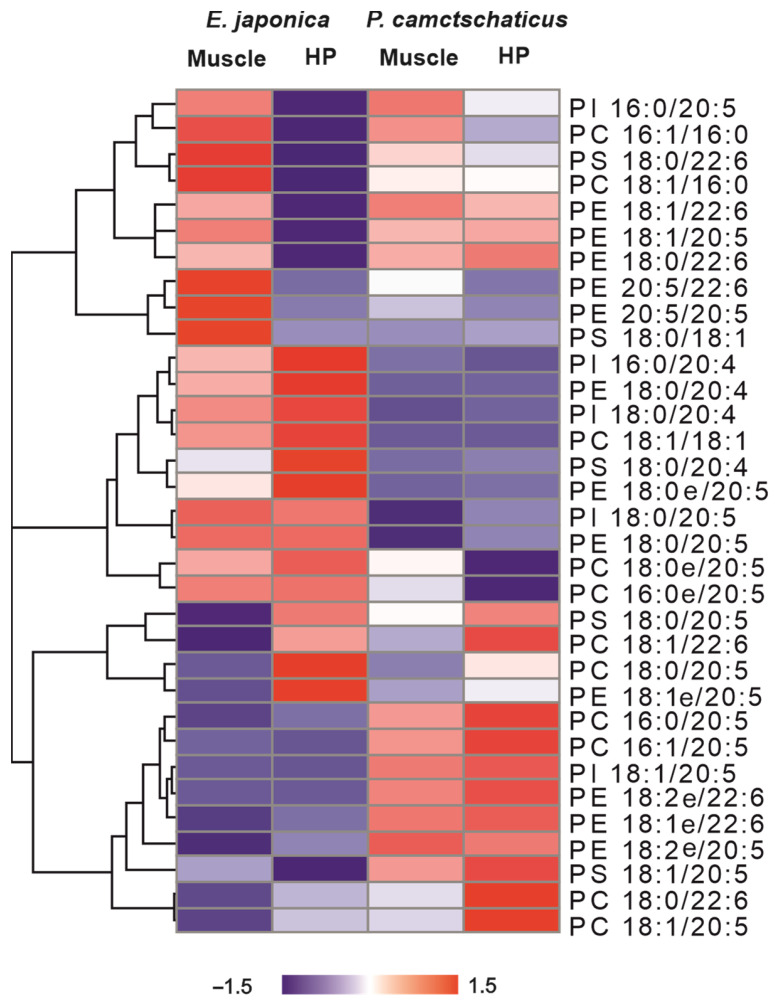
Heat maps of average data of main phospholipid molecular species with a clustering (tree clustering, wards method, and Euclidean distances). HP: hepatopancreas; PE: glycerophosphoethanolamines, PC: glycerophosphocholines; PI: glycerophosphoinositols; PS: glycerophosphoserines. The scale bar above the heatmap(s) represents the standard scaling to the relative abundance of lipid content (% of each class) in the samples.

The studied crabs had a number of distinguishing features in the composition of muscle and hepatopancreas phospholipids. A heat map of lipids was used to visualize the main differences in phospholipid composition of the studied samples of crabs (Figure 3). For heat map creation we used the content of PL molecular species (average data of % of each phospholipid class) based on the results of a two-way ANOVA and HSD test (*p* < 0.05) (Table S4). The Japanese mitten crab differed from the red king crab by a lower content of molecular species with 20:5 FA in PC, PS, and PI; an increased content of 20:4 FA in PE, PS, and PI; and a lower content of molecular species with 22:6 FA in PE the hepatopancreas and muscle.

## 4. Discussion

Lipids as energy sources and structural components of cell membranes are necessary **for** the normal growth and survival of aquatic animals. The development of analytical methods has led to an increase in studies on the composition of lipid molecular species in marine organisms. Changes in crabs’ lipidome have been shown to occur under different diets [16,17,24] and fungal infection [15,25]. We identified the profile molecular species of main lipid classes in the hepatopancreas and muscles of the crabs *E. japonica* and *P. camtschaticus*.

### 4.1. Differences in Lipid Composition between the Crabs E. japonica and P. camtschaticus

We determined 10 lipid classes in the hepatopancreases and muscles of *E. japonica* and *P. camtschaticus*. The hepatopancreases of the studied crabs contained mainly TGs (76.01% and 55.17% of all detected lipid classes for *E. japonica* and *P. camtschaticus*, respectively). As was described earlier, TGs also dominate edible viscera of *P. camtschaticus* and *Eriocheir sinensis* [1]. The major phospholipids in the hepatopancreas are PE and PC, which is confirmed by earlier studies [2,24].

In our study, TGs in the crab hepatopancreas were represented by a great amount of various molecular species. A total of 98 and 59 molecular species of TG were detected in *E. japonica* and *P. camtschaticus*, respectively. We found only nine of the same molecular species of TG in hepatopancreas lipids of *E. japonica* and *P. camtschaticus*. Xu et al. (2021) showed earlier a diversity of TG in the *E. sinensis* hepatopancreas [24]. Yao et al. (2023) detected various molecular species of TG in edible viscera of *P. camtschaticus* and *E. sinensis*. Authors showed that 16:0/20:1/18:2 and 16:0/18:1/20:1 were the main TGs in *P. camtschaticus* and that 16:0/16:0/20:5, 16:0/16:1/20:1, and 14:0/18:0/18:1 were a considerable part of *E. sinensis* edible viscera [1]. The TGs of *E. japonica* differed from those of *P. camtschaticus* by a higher content of 16:0, 16:1, 18:2, and 20:4 FA. Significant differences in the composition of TG were also found when compared with *E. sinensis* edible viscera [1]. *E. japonica* crabs spend and feed in freshwater habitats as a considerable part of their lives [18]. C_18:2_ FA is derived from freshwater plants [26]. It is known that TG is a primary storage compound in decapod crustaceans [27]. Such a diversity in TG molecular species may be associated with the significant differences between the diets of the studied crabs.

Muscles of the studied crabs contained a high amount of DGs, which was not encountered in previous studies. High amounts of DGs in the muscles and liver were reported earlier for the surgeonfish *Acanthurus bariene* [28]. Authors associated it with the action of phospholipases, but they could not identify any aspects of handling and processing that would produce the elevated values [28]. Lipids were extracted simultaneously from all our samples, and the hepatopancreas samples did not contain DG and MAG in the lipid extract despite the presence of active lipases [29]. Further investigation is needed to explain the role of the high DG content in crab muscles. Among the phospholipids, PC and PE were the main classes in the muscles of both crabs.

The major phospholipids of crab tissues were represented by the same molecular species. However, PE of the red king crab contained more DHA molecular species compared to the Japanese mitten crab. Phospholipids are more conservative compounds because they are constituents of cell and organelle membranes. However, the composition of crab phospholipids was shown to depend on diet [15]. The red king crab spends all its life in ocean or sea habitats, feeding on various marine organisms which can be additional sources of DHA. The profile of phospholipid molecular species of *E. japonica* and *E. sinensis* [1] is mostly similar. The main molecular species of PE, PC, and PI were the same. The phospholipids of *E. japonica* differed from *E. sinensis with* a higher content of C_20:4_-containg PI and lower content of C_22:6_-containig PE. We did not detect ether form of PI in *E. japonica* hepatopancreas in comparison to *E. sinensis* hepatopancreas [1]. The explanation for the differences in lipid profiles between these similar species needs clarification, since the Chinese mitten crab was farm raised, and we used wild Japanese mitten crab.

### 4.2. Nutritional Value of Crab Lipids

Diet is one of the major factors that may help prevent or promote a disease. Several health disorders including obesity, diabetes, cardiovascular diseases (atherosclerosis, hypertension, myocardial infarction, and stroke), osteoporosis, inflammatory disease, even infectious diseases, and certain cancers are associated with diet [13,30]. Marine lipids are characterized by high nutritional value and show various positive effects on human health [31]. The beneficial properties of marine lipids are associated mainly with a high n-3/n-6 PUFA ratio. The n-3/n-6 PUFA balance in the human diet is especially important for the treatment of inflammation. PUFA acts on inflammation through the production of oxylipins and the regulation of transcription factors and epigenetic changes [9]. The anti-inflammatory effect of n-3 PUFA on the immune response has been evidenced through human studies [32,33]. The number of studies considering the health-beneficial effects of PL and TG species esterified with n-3 PUFA from marine sources has increased recently [34].

Paluchova et al. identified triacylglycerol-based marine oil as an optimal nutritional source of DHA which supports production of anti-inflammatory fatty acid esters of hydroxy fatty acids [35]. In our study, TGs with EPA and DHA dominated the lipids of the red king crab hepatopancreas. As a reserve class of lipids, TGs are an available source of fatty acids for energy and structural needs. The crab hepatopancreas, used as an edible part, was characterized by a high content of lipids with the major class being TG. The red king crab hepatopancreas, which is rich in molecular species of triglycerides with EPA and DHA, can become a source of n-3 PUFA.

The muscles of the studied crabs were dominated by phospholipids containing mainly EPA and DHA. Currently, special attention is paid to the implication of n-3 PUFA-containing phospholipids in the human diet. The beneficial properties of EPA/DHA-PL are considered in multiple reviews [34,36,37,38]. The nutritional functions of EPA-PL include improvement in brain functions, visual development, nervous system development, and regulation of blood lipids [39]. The biological activities of DHA-PL comprise anti-neurodegeneration, anti-neuroinflammation, and anticancer; they also show benefits in the management of obesity and visual problems [40]. It has been reported that EPA- and DHA-PL significantly ameliorate depression-like behavior in mice [41].

The PE and PC of the studied crab tissues included molecular species with ether bond. We could not determine whether PE and PC have an alkyl or alkenyl bond. However, it was previously shown that ether-PE in *P. camtschaticus* lipids is present in a plasmalogen form [1]. Ether lipids are an important group of lipids characterized by an alkyl or alkenyl bond (plasmalogen) at the *sn*-1 position of the glycerol backbone. Ether phospholipids are among the major constituents of biological membranes; impairment in their biosynthesis leads to serious diseases (Rhizomelic Chondrodysplasia Punctata, Zellweger syndrome) [42]. A decreased level of plasmalogens (the most abundant form of ether-phospholipids in mammals) is associated with smoking-related lung disease and was also found in brains of Alzheimer’s and Parkinson’s disease patients [43]. It was recently reported that dietary supplementation with alkenylacyl PE and alkylacyl PC decreased the total cholesterol and low-density lipoprotein cholesterol concentrations in the serum of high-fat-induced atherosclerotic hamsters [44].

The study has shown that the crabs *E. japonica* and *P. camtschaticus* differ in the composition of TG and PL molecular species. Significant differences in the TG profile are probably caused by the specifics of crabs’ diet, whereas PL, as a structural component of cell membranes, is affected by genetic differences in crabs. Hepatopancreas of red king crab may be a source of TG containing n-3 PUFA. The muscles of the studied crabs contain predominantly molecular species of PL with n-3 PUFA, whose significance in the diet has received special attention of researchers. The data of the lipid molecular species profile provide new background information for future studies in the fields of biochemistry and aquaculture of the *E. japonica* and *P. camtschaticus* crabs in the Sea of Japan.

## Figures and Tables

**Table 1:** Lipid composition of muscle and hepatopancreas in the crabs *Eriocheir japonica* and *Paralithodes camtschaticus*.

Lipids	*E. japonicus*				*P. camchaticus*			
	Muscles		Hepatopancreas		Muscles		Hepatopancreas	
	mg/100 g w.w.	% of Detected Lipids	mg/100 g w.w.	% of Detected Lipids	mg/100 g w.w.	% of Detected Lipids	mg/100 g w.w.	% of Detected Lipids
TG	tr	tr	2405.46 ± 446.80	75.55 ± 7.05	tr	tr	460.26 ± 85.85	55.22 ± 0.52
DG	49.54 ± 10.99	26.32 ± 6.53	tr	tr	22.23 ± 9.43	20.58 ± 8.04	tr	tr
ST	2.81 ± 0.91	1.51 ± 0.57	246.15 ± 54.87	7.95 ± 2.65	1.44 ± 0.21	1.36 ± 0.21	24.75 ± 3.06	3.02 ± 0.50
PE	43.27 ± 7.95	22.79 ± 3.07	130.28 ± 14.05	4.17 ± 0.84	20.22 ± 5.08	18.90 ± 3.47	108.11 ± 24.67	12.92 ± 0.65
PC	60.76 ± 11.70	31.98 ± 4.43	353.21 ± 62.43	11.37 ± 3.26	37.04 ± 7.89	34.52 ± 0.40	203.05 ± 39.91	24.33 ± 0.34
PI	10.98 ± 0.65	5.82 ± 0.57	18.57 ± 4.97	0.60 ± 0.22	8.79 ± 1.38	8.27 ± 0.96	15.71 ± 5.19	1.85 ± 0.34
PS	6.95 ± 0.23	3.69 ± 0.35	5.58 ± 1.42	0.18 ± 0.06	7.68 ± 1.39	7.19 ± 0.49	11.42 ± 3.01	1.36 ± 0.15
SM	12.17 ± 2.11	6.41 ± 0.82	4.66 ± 2.04	0.15 ± 0.07	6.48 ± 2.74	6.12 ± 2.86	4.82 ± 1.47	0.57 ± 0.09
PA	2.65 ± 0.80	1.42 ± 0.51	tr	tr	1.79 ± 2.87	1.37 ± 2.13	tr	tr
LPE	tr	tr	0.56 ± 0.20	0.02 ± 0.01	1.61 ± 1.56	1.69 ± 1.72	5.70 ± 1.05	0.68 ± 0.03

Data are expressed as the mean ± SD (*n* = 4). TG: triacylglycerols; DG: diacylglycerols; ST: sterols; PE: glycerophosphoethanolamines; PC: glycerophosphocholines, PI: glycerophosphoinositols; PS: glycerophosphoserines; SM: sphingomyelins; PA: phosphatidic acids; LPE: lyso glecerophosphaethanolamines; tr: traces.

## Data Availability

Data is contained within the article or supplementary materials.

## Supplementary Materials

The following supporting information can be downloaded at: https://www.mdpi.com/article/10.3390/foods12183359/s1, Table S1: Amount of identified of lipid molecular species in muscle and hepatopancreas of crabs *Eriocheir japonica* and *Paralithodes camtschaticus*; Table S2: Profile of triacylglycerol molecular species of crab *Eriocheir japonica* hepatopancreas; Table S3: Profile of triacylglycerol and monoalkyldiacylglycerol molecular species of crab *Paralithodes camtschaticus* hepatopancreas; Table S4: The content of molecular species of glycerophosphoethanolamine, glycerophosphocholines, glycerophosphoserines, and glycerophosphoinositols in the different tissues (muscle and hepatopancreas) of different species crabs *Eriocheir japonica* and *Paralithodes camtschaticus* and the results of two-factor ANOVA; Table S5: Molecular species composition of sphingomyelins, phosphatidic acids and lyso-glycerophosphoethanolamine in in muscle and hepatopancreas of crabs *Eriocheir japonica* and *Paralithodes camtschaticus*; Figure S1: Heat maps of average data of main phospholipid molecular species with a clustering (tree clustering, wards method, and Euclidean distances).
