# Supplementary material for: Crabs Eriocheir japonica and Paralithodes camtschaticus Are a Rich Source of Lipid Molecular Species with High Nutritional Value
Source: Foods. 2023 Sep 7;12(18):3359. doi: 10.3390/foods12183359 (PMC10527590; doi:10.3390/foods12183359)
Supplement: Supplementary file 1 [file foods-12-03359-s001.zip › foods-2547106-supplementary.pdf]

Table S1: Amount of identified of lipid molecular species in muscle and hepatopancreas of crabs *Eriocheir japonica* and *Paralithodes camtschaticus*.

Table S2: Profile of triacylglycerol molecular species of crab *Eriocheir japonica* hepatopancreas

Table S3: Profile of triacylglycerol and monoalkyldiacylglycerol molecular species of crab *Paralithodes camtschaticus* hepatopancreas.

Table S4: The content of molecular species of glycerophosphoethanolamine, glycerophosphocholines, glycerophosphoserines, and glycerophosphoinositols in the different tissues (muscle and hepatopancreas) of different species crabs *Eriocheir japonica* and *Paralithodes camtschaticus* and the results of two-factor ANOVA

Table S5: Molecular species composition of sphingomyelins, phosphatidic acids and lyso-glycerophosphoethanolamine in in muscle and hepatopancreas of crabs *Eriocheir japonica* and *Paralithodes camtschaticus*

Table S1. Amount of identified of lipid molecular species in muscle and hepatopancreas of crabs *Eriocheir japonica* and *Paralithodes camtschaticus*

**Lipid class**	***E. japonica***		***P. camchaticus***	
	**Muscle**	**Hepatopancreas**	**Muscle**	**Hepatopancreas**
TG	nd	98	nd	59
DG	11	nd	5	nd
PE	33	28	34	32
PC	48	46	49	45
PS	15	3	11	10
PI	17	13	17	13
SM	7	3	10	7
PA	15	1	18	5
LPE	5	4	8	7
Total	151	196	152	178

Nd: not determined; TG: triacylglycerols; DG: diacylglycerols; PE: glycerophosphoethanolamines; PC: glycerophosphocholines, PS: glycerophosphoserines; PI: glycerophosphoinositols; SM: sphingomyelins; PA: phosphatidic acids; LPE: lyso glecerophosphaethanolamines

Table S2. Profile of triacylglycerol molecular species of crab *Eriocheir japonica* hepatopancreas

**Molecular species**	**Formula**	**[M+NH4]+**	**Content, % of TG sum**
18:1/16:0/20:0*	C57 H108 O6	906.8411	2.34±0.64
18:1/16:0/18:1	C55 H102 O6	876.8014	8.79±5.42
16:1/16:0/18:0; 16:0/16:0/18:1	C53 H100 O6	850.785	3.63±1.63
16:0/16:0/16:0; 18:0/16:0/14:0; 18:1/16:0/20:3	C51 H98 O6; C57 H102 O6	824.7765; 900.8005	3.26±0.71
18:1/18:1/18:2**; 16:1/18:1/20:2	C57 H102 O6	900.7982	3.18±0.57
18:1/16:0/18:2	C55 H100 O6	874.785	5.74±1.21
20:4/16:0/18:1; 18:1/16:1/18:1	C57 H100 O6; C55 H100 O6	898.782; 874.785	7.63±1.28
16:1/14:0/16:1	C53 H98 O6	848.775	9.82±0.72
16:1/16:0/16:0; 16:1/14:0/18:0;	C51 H96 O6	822.757	6.07±0.42

18:1/14:0/16:0			
20:5/18:1/18:1;<br>20:3/16:1/18:1;<br>18:3/18:1/16:0;<br>16:0/16:0/14:0	C59 H100 O6;<br>C57 H100 O6;<br>C55 H98 O6;<br>C49 H94 O6	922.7865;<br>898.7846;<br>872.7703;<br>796.7377	2.37±0.38
20:5/18:1/16:0	C57 H98 O6	896.771	6.06±0.51
16:1/16:0/17:1;<br>18:1/31:1;<br>18:1/16:1/18:2	C52 H96 O6;<br>C55 H98 O6	834.7513;<br>872.77	3.96±0.66
18:2/16:0/16:1;<br>16:2/16:0/18:1;<br>22:5/16:0/16:1;<br>20:4/34:2;<br>18:2/16:0/16:1	C53 H96 O6;<br>C57 H98 O6	846.7586<br>;896.7692	3.36±0.83
20:4/16:0/16:1;<br>22:5/14:0/16:0;<br>16:1/16:1/18:1	C55 H96 O6;<br>C53 H96 O6	870.7565;<br>846.756	6.06±2.12
20:5/18:1/18:2;<br>16:1/16:0/16:1;<br>18:3/16:0/18:2	C59 H98 O6;<br>C51 H94 O6;<br>C55 H96 O6	920.7706;<br>820.7386;<br>870.7559	4.85±2.21
20:5/18:2/16:0;<br>18:3/16:1/18:1;<br>16:1/16:0/14:0;<br>20:5/16:1/18:1;<br>18:3/16:1/16:0	C57 H96 O6;<br>C55 H96 O6;<br>C49 H92 O6;<br>C57 H96 O6;<br>C53 H94 O6	894.759;<br>870.7576;<br>794.7246;<br>894.7565;<br>844.7413	6.06±2.78
20:5/14:0/18:1;<br>20:5/16:0/16:1	C55 H94 O6	868.7737	3.18±1.53
20:5/16:0/14:0;<br>16:1/18:2/16:1;<br>20:5/36:4; 22:6/34:3	C53 H92 O6;<br>C53 H94 O6;<br>C59 H96 O6	842.7244;<br>844.7397;<br>918.7538	1.24±0.88
16:1/14:0/18:2;<br>20:5/16:0/18:3;<br>20:4/16:1/16:1	C51 H92 O6;<br>C57 H94 O6;<br>C55 H94 O6	818.7237;<br>892.7392;<br>868.7396	1.39±0.80
20:5/18:1/18:4;<br>16:1/14:1/18:1;	C59 H94 O6;<br>C51 H92 O6;	916.7395;<br>818.721;	2.07±1.39

18:3/16:1/18:2;<br>20:4/30:1;<br>16:3/34:2	C55 H94 O6;<br>C53 H92 O6	868.867;<br>842.725	
20:5/16:1/18:2;<br>16:1/16:0/14:1;<br>18:2/14:0/14:0	C57 H94 O6;<br>C49 H90 O6	892.7397;<br>792.709	0.50±0.41
20:5/16:1/20:4;<br>20:5/16:1/16:1;<br>22:6/30:1;<br>16:4/36:3;<br>16:1/14:0/16:1;<br>18:1/14:0/14:1;<br>16:3/16:1/18:1;<br>18:3/14:0/18:2	C59 H94 O6;<br>C55 H92 O6;<br>C49 H90 O6;<br>C53 H92 O6	916.739;<br>866.7246;<br>792.7098;<br>842.7232	0.57±0.42
16:1/16:1/18:3;<br>16:3/32:1;<br>20:5/14:0/18:2;<br>16:1/14:0/14:0;<br>14:1/16:0/14:0;<br>18:1/14:0/12:0	C53 H92 O6;<br>C51 H90 O6;<br>C55 H92 O6;<br>C47 H88 O6	842.725;<br>816.7144;<br>866.7246;<br>766.6948	0.47±0.43
20:5/16:1/16:1	C55 H92 O6	866.7246	0.74±0.52
18:3/14:0/16:1	C51 H90 O6	816.7144	0.25±0.21
16:1/16:2/16:1;<br>20:5/14:0/16:1	C51 H90 O6;<br>C53 H90 O6	816.7144;<br>840.7077	0.32±0.26
16:4/32:1;<br>20:5/32:3;<br>20:5/34:4	C51 H88 O6;<br>C55 H90 O6;<br>C57 H92 O6	814.6919;<br>864.7081;<br>890.7204	0.19±0.15
14:0/16:1/16:2;<br>20:5/28:0;<br>18:4/16:1/16:1;<br>16:3/18:2/16:1;<br>20:5/16:1/20:5	C49 H88 O6;<br>C51 H88 O6;<br>C53 H90 O6;<br>C59 H92 O6	790.6907;<br>814.6919;<br>840.7078;<br>914.7235	0.20±0.18
20:5/16:2/16:1;<br>20:4/32:4;<br>16:1/16:1/14:1;	C55 H90 O6;<br>C49 H88 O6;<br>C57 H90 O6	864.7096;<br>790.6891;<br>888.7078	0.20±0.20

20:5/14:0/20:5; 20:5/14:1/20:4			
16:4/34:3; 20:5/30:4; 16:3/16:1/16:1; 20:5/14:0/20:5	C53 H88 O6; C51 H88 O6; C57 H90 O6	838.6918;814.6919; 888.7078	0.11±0.10
20:5/14:1/16:1; 16:3/16:1/14:0	C53 H88 O6; C49 H86 O6	838.6918; 788.6792	0.06±0.04
20:5/16:0/16:4; 20:5/16:1/16:3; 16:4/32:2; 16:3/16:3/16:1	C55 H88 O6; C51 H86 O6; C53 H88 O6	862.6945; 812.6781; 838.6918	0.07±0.08
Other			5.26±4.82
TG sum			

Data were presented as mean ± SD, *n* = 4

*TG – *sn*-1(3)/*sn*-2/*sn*-3(1); **Green color indicated undetermined *sn*-position of acyl chains

Table S3. Profile of triacylglycerol and monoalkyldiacylglycerol molecular species of crab *Paralithodes camtschaticus* hepatopancreas

Molecular species	Formula	$[M+NH_4]^+$	Content, % of TG sum
18:1/18:1/20:1*; 22:6/20:1/20:1	C59H108O6; C65H110O6	930.8443; 1004.8627	2.08±0.68
20:1/18:1/16:0	C57H106O6	904.83	2.17±0.80
20:5/20:1/20:1; 16:0/20:1/16:0; 18:0/16:0/18:1; 20:5/36:1alk	C63H108O6; C55H104O6; C59H104O5	978.8479; 878.8157; 910.8187	1.19±0.47
20:5/20:1/20:1; 24:6/20:1/16:0**; 20:5/18:1/22:1	C63H108O6	978.85	1.60±0.28
20:5/20:1/18:0; 20:5/18:1/20:0	C61H106O6	952.83	1.02±0.05
18:1/15:0/20:1; 20:2/16:1/20:1	C56H104O6; C59H106O6	890.8085; 928.8222	1.02±0.39

20:4/18:1/20:1	C61H106O6	952.83	1.73±0.46
18:1/18:0/16:1; 16:1/20:1/16:0; 22:6/18:1/18:0	C55H102O6; C61H104O6	876.8002; 950.815	6.19±1.10
20:5/18:1/20:1	C61H104O6	950.81	2.59±0.87
20:5/20:1/18:1	C61H104O6	950.8134	6.44±0.67
20:5/18:1/18:0; 20:5/20:1/16:0	C59H102O6	924.7995	3.94±0.09
20:3/16:0/18:2; 20:2/16:1/18:1	C57H100O6; C57H102O6	898.7848; 900.7984	1.67±0.26
20:4/36:2; 18:4/34:2	C59H102O6	924.7987	2.25±0.52
22:6/18:1/18:1; 18:1/18:1/16:1; 16:1/16:1/20:1	C61H102O6; C55H100O6	948.8000; 874.7817	8.67±0.54
22:6/16:0/18:1; 20:5/18:1/20:1; 18:1/16:0/16:1; 18:1/18:1/14:0	C59H100O6; C53H98O6	922.7811; 848.7666	6.68±0.34
20:5/16:1/20:1	C59H100O6	922.78	1.34±0.11
20:5/18:1/18:1	C59H100O6	922.78	10.21±0.50
20:5/16:0/18:1	C57H98O6	896.77	5.17±0.85
20:5/16:0/16:0; 18:2/16:0/18:3; 16:2/36:3	C55H96O6	870.75	2.02±0.42
20:5/20:1/20:5	C63H100O6; C57H98O6	970.7826; 896.7688	3.42±0.34
16:1/16:1/18:1; 22:6/18:1/16:1; 20:5/18:0/20:5; 20:4/32:1; 18:4/34:1	C53H96O6; C59H98O6; C61H98O6; C55H96O6	846.7548; 920.7671; 944.7662; 870.7461	4.63±0.67
20:5/18:1/20:4; 18:1/16:1/14:0; 16:1/16:1/16:0;	C61H98O6; C51H94O6; C57H96O6	944.7648; 820.738; 894.7487	2.66±0.48

22:6/16:0/16:1			
20:5/16:1/18:1;<br>20:5/18:1/22:6	C57H96O6;<br>C63H98O6	894.7487;<br>968.7666	7.11±1.22
20:5/16:0/16:1	C55H94O6	868.7345	2.65±0.53
20:5/18:1/20:5	C61H96O6	942.7448	3.75±0.87
20:5/16:0/20:5	C59H94O6	916.7354	1.65±0.52
20:5/18:1/20:4	C59H94O6	916.7354	0.71±0.28
20:5/16:1/16:1	C55H92O6	866.7187	1.25±0.33
20:5/30:1	C53H90O6	840.7059	0.50±0.14
20:5/16:1/20:5	C59H92O6	914.7167	1.51±0.34
Other			2.18±0.43
TG sum			

*,** See Table 1

Table S4. Molecular species composition of sphingomyelins, phosphatidic acids and lyso- glycer-ophosphoethanolamine in in muscle and hepatopancreas of crabs *Eriocheir japonica* and *Paralithodes camtschaticus*

Molecular species	Formula	Detected *m/z*	*Eriocheir japonica*		*Paralithodes camtschaticus*		Factors			
			Muscle	Hepatopancreas	Muscle	Hepatopancreas	F1,23, p	Species	Tissue	Species×Tissue
Phosphatidylethanolamines (% from total PE)										
		[M-H]$^-$								
PE 16:0/16:1[1]	C37 H72 N O8 P	688.4923	0.89±0.24	0.00±0.00	0.57±0.80	0.04±0.02	F	0.34	8.53	0.56
							p	0.58	0.02	0.47
PE±16:1e/18:1[2]	C39 H76 N O7 P	700.5287	1.24±0.43	1.44±0.09	0.10±0.09	0.02±0.01	F	99.45	0.18	1.13
							p	0.00	0.68	0.32
PE 34:2	C39 H74 N O8 P	714.5079	1.53±0.46	0.38±0.29	0.14±0.10	0.02±0.02	F	29.45	15.71	10.33
							p	0.00	0.00	0.01
PE 16:0/18:1	C39 H76 N O8 P	716.5236	1.25±0.15	0.06±0.05	0.14±0.18	0.00±0.00	F	68.99	88.59	55.48
							p	0.00	0.00	0.00
PE 16:1e/20:5	C41 H72 N O7 P	720.4974	2.89±0.99	7.03±1.37	5.19±0.51	4.95±0.17	F	0.04	14.40	18.22
							p	0.84	0.01	0.00
PE 16:1e/20:4	C41 H74 N O7 P	722.513	2.60±1.10	3.33±2.31	1.54±0.15	1.44±0.12	F	3.97	0.18	0.32

							p	0.08	0.68	0.59
PE 18:1e/18:1	C41 H80 N O7 P	728.56	0.39±0.07	0.55±0.19	0.11±0.04	0.00±0.00	F	47.68	0.17	4.69
							p	0.00	0.69	0.06
PE 17:1e/20:5	C42 H74 N O7 P	734.513	1.16±0.21	3.03±0.80	1.42±0.18	1.30±0.10	F	9.03	12.66	16.37
							p	0.02	0.01	0.00
PE 16:1/20:5	C41 H70 N O8 P	734.4766	0.88±0.22	0.18±0.08	1.61±0.63	1.41±0.08	F	25.08	5.31	1.64
							p	0.00	0.05	0.24
PE 17:0e/20:5	C42 H76 N O7 P	736.5287	0.50±0.05	0.84±0.30	0.41±0.14	0.25±0.05	F	11.56	0.84	6.45
							p	0.01	0.39	0.03
PE 16:0/20:5	C41 H72 N O8 P	736.4923	3.38±0.45	1.95±0.41	3.16±1.08	2.50±0.25	F	0.21	8.22	1.10
							p	0.66	0.02	0.32
PE 16:0/20:4	C41 H74 N O8 P	738.5079	0.93±0.31	1.56±0.53	0.49±0.21	0.63±0.06	F	13.20	4.06	1.72
							p	0.01	0.08	0.23
PE 18:1/18:1	C41 H78 N O8 P	742.5392	1.62±0.14	0.71±0.19	0.21±0.07	0.08±0.04	F	199.87	51.58	28.54
							p	0.00	0.00	0.00
PE 18:2e/20:5	C43 H74 N O7 P	746.513	3.97±0.74	5.38±0.35	9.54±0.86	8.93±0.51	F	148.46	1.17	7.26
							p	0.00	0.31	0.03
PE 18:1e/20:5	C43 H76 N O7 P	748.5287	10.55±0.87	20.01±3.84	12.49±0.72	14.02±0.52	F	3.02	22.28	11.62
							p	0.12	0.00	0.01
PE 18:0e/20:5	C43 H78 N O7 P	750.5443	5.36±0.59	9.69±2.42	2.31±0.02	2.63±0.77	F	45.06	9.51	7.09
							p	0.00	0.02	0.03
PE 17:0/20:5	C42 H74 N O8 P	750.5079	0.94±0.24	1.01±0.05	0.69±0.06	0.78±0.11	F	9.13	1.04	0.01
							p	0.02	0.34	0.94
PE 17:1e/22:6	C44 H76 N O7 P	760.5287	0.66±0.09	0.96±0.46	1.00±0.12	1.00±0.12	F	1.75	1.15	1.14
							p	0.22	0.32	0.32
PE 18:2/20:5	C43 H72 N O8 P	760.4923	2.86±0.14	0.51±0.17	1.88±0.52	1.55±0.43	F	0.02	43.00	24.09
							p	0.89	0.00	0.00
PE 17:0e/22:6	C44 H78 N O7 P	762.5443	1.11±0.06	1.56±0.39	1.02±0.13	1.03±0.10	F	6.20	3.54	3.25
							p	0.04	0.10	0.11
PE 18:1/20:5	C43 H74 N O8 P	762.5079	14.06±1.48	9.25±1.56	13.23±0.73	13.47±1.66	F	4.38	7.93	9.65
							p	0.07	0.02	0.01
PE 18:0/20:5	C43 H76 N O8 P	764.5236	12.20±1.06	12.22±2.45	7.11±2.23	8.47±0.47	F	19.08	0.47	0.44
							p	0.00	0.51	0.53
PE 18:0/20:4	C43 H78 N O8 P	766.5392	3.09±0.51	4.52±1.42	0.88±0.39	0.95±0.13	F	41.08	2.78	2.25

							p	0.00	0.13	0.17
PE 18:2e/22:6	C45 H76 N O7 P	772.5287	0.64±0.06	0.67±0.23	2.09±0.82	2.46±0.26	F	39.70	0.56	0.44
							p	0.00	0.48	0.53
PE 18:1e/22:6	C45 H78 N O7 P	774.5443	3.83±0.19	5.17±1.39	11.98±0.59	12.82±1.03	F	220.76	4.24	0.22
							p	0.00	0.07	0.65
PE 18:0e/22:6	C45 H80 N O7 P	776.56	3.18±0.33	5.32±1.51	4.65±1.12	5.41±0.88	F	1.66	5.69	1.27
							p	0.23	0.04	0.29
PE 17:0/22:6	C44 H76 N O8 P	776.5236	0.82±0.14	0.25±0.05	0.82±0.07	0.59±0.08	F	10.67	61.97	11.28
							p	0.01	0.00	0.01
PE 20:5/20:5	C45 H70 N O8 P	782.4766	3.24±1.29	0.00±0.00	0.57±0.47	0.06±0.06	F	10.75	22.17	11.77
							p	0.01	0.00	0.01
PE 18:1/22:6	C45 H76 N O8 P	788.5236	6.76±0.91	1.24±0.34	7.49±2.36	6.50±1.26	F	13.30	15.66	7.63
							p	0.01	0.00	0.02
PE 18:0/22:6	C45 H78 N O8 P	790.5392	3.62±0.15	1.18±0.52	3.68±0.67	4.12±0.41	F	29.50	13.19	27.32
							p	0.00	0.01	0.00
PE 20:2e/22:6	C47 H80 N O7 P	800.56	0.00±0.00	0.00±0.00	1.29±0.44	1.60±0.32	F	84.68	0.98	0.98
							p	0.00	0.35	0.35
PE 19:1/22:6	C46 H78 N O8 P	802.5392	0.08±0.01	0.00±0.00	0.39±0.22	0.29±0.14	F	15.62	1.50	0.04
							p	0.00	0.26	0.85
PE 20:5/22:6	C47 H72 N O8 P	808.4923	3.58±1.22	0.00±0.00	1.18±1.56	0.12±0.02	F	3.97	16.49	4.86
							p	0.08	0.00	0.06
PE 20:1/22:6	C47 H80 N O8 P	816.5549	0.17±0.16	0.00±0.00	0.63±0.04	0.59±0.04	F	113.26	4.90	1.74
							p	0.00	0.06	0.22
ether PE			38.09±3.51	64.97±6.38	55.14±1.37	57.85±3.73	F	4.30	38.23	25.48
							p	0.07	0.00	0.00
C20:5 PE			61.99±2.12	71.11±1.72	59.61±1.64	60.31±0.89	F	47.55	26.41	19.43
							p	0.00	0.00	0.00
C22:6 PE			24.46±1.99	16.34±3.81	36.21±1.17	36.52±0.87	F	148.73	8.90	10.34
							p	0.00	0.02	0.01
C20:4 PE			6.62±1.90	9.41±4.05	2.91±0.68	3.01±0.02	F	14.99	1.23	1.06
							p	0.00	0.30	0.33
Phosphatidylcholines (% from total PC)										
		$[M+HCOOH]^-$								

PC 33:1	C39 H76 N O8 P	762.5291	0.35±0.14	0.15±0.05	1.16±0.63	1.40±0.39	F	22.44	0.01	1.03
							p	0.00	0.94	0.34
PC 34:2e	C40 H80 N O7 P	762.5654	1.38±0.29	0.77±0.05	0.12±0.15	0.00±0.00	F	113.16	14.73	6.63
							p	0.00	0.00	0.03
PC 32:2	C40 H76 N O8 P	774.5291	0.61±0.14	0.41±0.29	0.89±0.23	0.57±0.22	F	2.83	4.04	0.25
							p	0.13	0.08	0.63
PC 16:1/16:0	C40 H78 N O8 P	776.5447	3.14±0.29	1.93±0.22	2.90±0.96	2.38±0.25	F	0.11	8.08	1.30
							p	0.74	0.02	0.29
PC 33:1e	C41 H82 N O7 P	776.5811	1.08±0.10	0.85±0.41	0.73±0.40	0.27±0.05	F	7.67	4.19	0.50
							p	0.02	0.07	0.50
PC 32:0	C40 H80 N O8 P	778.5604	0.19±0.18	0.31±0.09	0.16±0.08	0.14±0.09	F	2.30	0.50	1.05
							p	0.17	0.50	0.34
PC 34:2e	C42 H82 N O7 P	788.5811	3.40±1.07	1.55±0.03	0.58±0.49	0.00±0.00	F	41.15	12.75	3.47
							p	0.00	0.01	0.10
PC 33:1	C41 H80 N O8 P	790.5604	2.22±0.32	2.60±0.23	2.34±0.36	2.51±0.17	F	0.01	2.92	0.40
							p	0.93	0.13	0.54
PC 34:1e	C42 H84 N O7 P	790.5967	5.30±2.48	1.60±0.16	0.42±0.26	0.07±0.04	F	19.80	7.92	5.44
							p	0.00	0.02	0.05
PC 34:3	C42 H78 N O8 P	800.5447	1.88±0.23	1.16±1.08	0.69±0.40	0.13±0.07	F	10.55	3.48	0.06
							p	0.01	0.10	0.82
PC 34:2	C42 H80 N O8 P	802.5604	4.56±0.19	5.57±0.95	2.89±0.52	3.47±0.40	F	31.07	5.48	0.41
							p	0.00	0.05	0.54
PC 18:1/16:0	C42 H82 N O8 P	804.576	10.49±0.93	6.17±0.41	8.57±2.55	8.44±0.85	F	0.05	7.17	6.36
							p	0.84	0.03	0.04
PC 18:0e/17:1	C43 H86 N O7 P	804.6124	0.43±0.09	0.17±0.10	0.53±0.00	0.21±0.07	F	2.51	43.98	0.45
							p	0.15	0.00	0.52
PC 36:6e	C44 H78 N O7 P	808.5498	0.78±0.03	1.03±0.16	1.17±0.56	0.87±0.02	F	0.51	0.02	2.63
							p	0.50	0.90	0.14
PC 16:0e/20:5	C44 H80 N O7 P	810.5654	4.01±0.85	4.07±0.66	3.21±0.96	2.05±0.11	F	11.26	1.72	2.14
							p	0.01	0.23	0.18
PC 36:4e	C44 H82 N O7 P	812.5811	1.08±0.29	1.53±0.84	0.42±0.22	0.12±0.01	F	15.39	0.08	2.04
							p	0.00	0.79	0.19
PC 35:3	C43 H82 N O8 P	816.576	0.75±0.11	1.31±0.24	0.61±0.13	0.67±0.09	F	19.44	11.98	7.79
							p	0.00	0.01	0.02

PC 36:2e	C44 H86 N O7 P	816.6124	0.73±0.11	0.12±0.11	0.00±0.00	0.00±0.00	F	87.36	45.99	45.99
							p	0.00	0.00	0.00
PC 18:0/17:1	C43 H84 N O8 P	818.5917	1.77±0.16	1.75±0.15	1.12±0.46	1.03±0.34	F	14.87	0.11	0.03
							p	0.00	0.75	0.86
PC 16:1/20:5	C44 H76 N O8 P	822.5291	1.24±0.27	0.99±0.13	5.50±1.77	7.33±1.88	F	49.87	1.10	1.91
						±	p	0.00	0.32	0.20
PC 16:0/20:5	C44 H78 N O8 P	824.5447	4.19±0.27	5.16±0.53	9.33±1.09	11.38±1.28	F	121.38	8.56	1.12
							p	0.00	0.02	0.32
PC 37:5e	C45 H82 N O7 P	824.5811	0.98±0.20	1.18±0.36	0.87±0.31	0.41±0.04	F	8.72	0.75	4.77
							p	0.02	0.41	0.06
PC 36:4	C44 H80 N O8 P	826.5604	0.99±0.58	2.32±1.25	1.08±0.55	1.34±0.21	F	1.05	3.35	1.52
							p	0.33	0.10	0.25
PC 36:3	C44 H82 N O8 P	828.576	2.52±0.05	3.42±0.92	0.60±0.49	0.00±0.00	F	78.79	0.24	6.23
							p	0.00	0.64	0.04
PC 18:1/18:1	C44 H84 N O8 P	830.5917	5.53±0.19	6.49±1.20	3.09±0.67	3.07±0.28	F	51.50	1.33	1.42
							p	0.00	0.28	0.27
PC 18:1/18:0	C44 H86 N O8 P	832.6073	3.21±0.89	1.28±0.28	2.01±1.11	1.25±0.38	F	2.02	9.69	1.82
							p	0.19	0.01	0.21
PC 37:6	C45 H78 N O8 P	836.5447	0.38±0.26	0.13±0.06	0.77±0.21	0.81±0.15	F	25.01	0.95	1.80
							p	0.00	0.36	0.22
PC 16:0e/22:6	C46 H82 N O7 P	836.5811	2.31±0.30	4.35±0.73	3.04±0.30	2.67±0.26	F	3.52	10.79	22.57
							p	0.10	0.01	0.00
PC 17:0/20:5	C45 H80 N O8 P	838.5604	0.67±0.08	1.44±0.23	0.86±0.12	1.27±0.15	F	0.02	43.09	4.30
							p	0.89	0.00	0.07
PC 18:0e/20:5	C46 H84 N O7 P	838.5967	3.90±0.37	4.38±0.22	3.51±0.79	2.11±0.07	F	26.32	3.07	13.10
							p	0.00	0.12	0.01
PC 38:4e	C46 H86 N O7 P	840.6124	0.97±0.06	0.94±0.08	0.42±0.44	0.06±0.07	F	29.45	2.12	1.62
							p	0.00	0.18	0.24
PC 38:7	C46 H78 N O8 P	848.5447	2.44±0.25	0.94±0.11	1.97±0.44	1.77±0.08	F	1.36	31.24	18.26
							p	0.28	0.00	0.00
PC 18:1/20:5	C46 H80 N O8 P	850.5604	7.48±0.60	9.62±1.56	9.92±2.42	14.65±1.30	F	16.23	13.75	1.94
							p	0.00	0.01	0.20
PC 18:0/20:5	C46 H82 N O8 P	852.576	3.28±0.23	8.91±2.07	3.89±1.68	5.76±0.59	F	2.58	22.54	5.68
							p	0.15	0.00	0.04

PC 18:0/20:4	C46 H84 N O8 P	854.5917	1.12±0.48	2.26±1.04	0.63±0.29	0.70±0.08	F	8.98	3.19	2.47
							p	0.02	0.11	0.15
PC 38:3	C46 H86 N O8 P	856.6073	0.38±0.22	0.71±0.51	0.18±0.09	0.00±0.00	F	7.83	0.23	2.46
							p	0.02	0.65	0.16
PC 38:2	C46 H88 N O8 P	858.623	0.64±0.23	0.61±0.41	0.85±0.48	0.93±0.17	F	1.73	0.01	0.07
							p	0.22	0.91	0.79
PC 40:7e	C48 H84 N O7 P	862.5967	0.30±0.27	0.78±0.50	1.46±0.75	1.32±0.08	F	9.69	0.37	1.28
							p	0.01	0.56	0.29
PC 39:6	C47 H82 N O8 P	864.576	0.30±0.15	0.59±0.11	0.62±0.20	1.09±0.11	F	23.14	20.25	1.04
							p	0.00	0.00	0.34
PC 20:1e/20:5	C48 H86 N O7 P	864.6124	0.97±0.33	1.98±0.46	4.52±0.67	3.32±0.56	F	65.86	0.10	13.51
							p	0.00	0.76	0.01
PC 40:5e	C48 H88 N O7 P	866.628	0.58±0.13	0.79±0.34	0.76±0.12	0.49±0.14	F	0.25	0.06	4.14
							p	0.63	0.81	0.08
PC 20:5/20:5	C48 H76 N O8 P	870.5291	3.11±0.82	0.00±0.00	3.97±2.95	0.61±0.12	F	0.68	13.37	0.02
							p	0.43	0.01	0.89
PC 40:9	C48 H78 N O8 P	872.5447	1.41±0.09	0.16±0.03	0.94±0.48	0.31±0.07	F	1.25	42.99	4.73
							p	0.30	0.00	0.06
PC 40:8	C48 H80 N O8 P	874.5604	1.07±0.11	0.30±0.02	0.52±0.45	0.03±0.03	F	9.32	21.50	1.13
							p	0.02	0.00	0.32
PC 18:1/22:6	C48 H82 N O8 P	876.576	1.61±0.12	3.06±0.21	2.32±0.67	3.55±0.40	F	6.49	32.37	0.21
							p	0.03	0.00	0.66
PC 18:0/22:6	C48 H84 N O8 P	878.5917	1.33±0.30	2.34±0.81	2.64±1.58	5.09±0.55	F	13.90	10.09	1.74
							p	0.01	0.01	0.22
PC 40:5	C48 H86 N O8 P	880.6073	1.11±0.28	1.81±0.05	1.19±0.40	1.19±0.53	F	1.69	2.78	2.79
							p	0.23	0.13	0.13
PC 20:2e/22:5	C50 H88 N O7 P	890.628	0.00±0.00	0.00±0.00	1.19±0.70	1.12±0.28	F	28.46	0.02	0.02
							p	0.00	0.89	0.89
PC 20:5/22:6	C50 H78 N O8 P	896.5447	1.80±0.11	0.00±0.00	1.83±1.04	0.46±0.09	F	0.65	27.05	0.52
							p	0.44	0.00	0.49
PC 42:7	C50 H86 N O8 P	904.6073	0.00±0.00	0.00±0.00	0.98±0.56	1.53±0.41	F	39.79	1.94	1.94
							p	0.00	0.20	0.20
ether PC			28.19±3.57	26.09±3.13	22.96±1.80	15.11±1.39	F	28.49	10.73	3.57

							p	0.00	0.01	0.10
C20:5 PC			30.66±1.63	36.56±3.40	46.55±4.15	48.95±1.30	F	72.43	6.25	1.12
							p	0.00	0.04	0.32
C22:6 PC			7.06±0.53	9.76±1.60	9.84±1.94	11.78±1.29	F	8.35	7.80	0.21
							p	0.02	0.02	0.66
C20:4 PC			1.12±0.48	2.26±1.04	0.63±0.29	0.70±0.08	F	8.98	3.19	2.47
							p	0.02	0.11	0.15
Phosphatidylserines (% from total PS)										
		$[M-H]^-$								
PS 16:0/20:5	C42 H72 N O10 P	780.4821	0.84±0.34	0.00±0.00	1.35±0.92	0.45±0.50	F	2.22	7.52	0.01
							p	0.17	0.03	0.94
PS 18:0/18:1	C42 H80 N O10 P	788.5447	1.91±0.60	0.00±0.00	0.00±0.00	0.09±0.08	F	27.67	27.67	33.08
							p	0.00	0.00	0.00
PS 17:0/20:5	C43 H74 N O10 P	794.4978	0.47±0.26	0.00±0.00	2.03±0.35	1.36±0.91	F	24.92	3.88	0.11
							p	0.00	0.08	0.74
PS 18:1/20:5	C44 H74 N O10 P	806.4978	7.32±1.59	2.03±3.52	14.10±1.27	17.85±0.67	F	91.63	0.47	14.95
							p	0.00	0.51	0.00
PS 18:0/20:5	C44 H76 N O10 P	808.5134	24.24±2.36	50.67±10.57	41.90±3.33	49.89±2.55	F	6.00	25.40	7.19
							p	0.04	0.00	0.03
PS 18:0/20:4	C44 H78 N O10 P	810.5291	21.82±1.35	47.30±10.87	11.34±0.75	13.26±0.28	F	44.91	18.86	14.10
							p	0.00	0.00	0.01
PS 17:0/22:6	C45 H76 N O10 P	820.5134	1.47±0.77	0.00±0.00	2.65±1.95	0.00±0.00	F	0.94	11.72	0.94
							p	0.36	0.01	0.36
PS 19:0/20:5	C45 H78 N O10 P	822.5291	1.09±0.22	0.00±0.00	1.75±1.16	0.88±0.88	F	3.25	5.39	0.07
							p	0.11	0.05	0.79
PS 20:5/20:5	C46 H70 N O10 P	826.4665	0.11±0.11	0.00±0.00	0.00±0.00	0.00±0.00	F	3.34	3.34	3.34
							p	0.11	0.11	0.11
PS 18:1/22:6	C46 H76 N O10 P	832.5134	6.07±0.93	0.00±0.00	6.53±3.56	3.68±0.60	F	3.53	17.27	2.32
							p	0.10	0.00	0.17
PS 18:0/22:6	C46 H78 N O10 P	834.5291	23.30±1.46	0.00±0.00	13.67±1.64	10.59±3.74	F	0.08	113.54	67.06
							p	0.79	0.00	0.00
PS 18:0/22:5	C46 H80 N O10 P	836.5447	3.28±2.10	0.00±0.00	4.10±2.08	1.96±0.76	F	2.41	9.57	0.45
							p	0.16	0.01	0.52

PS 20:5/22:6	C48 H72 N O10 P	852.4821	3.64±0.92	0.00±0.00	0.00±0.00	0.00±0.00	F	41.07	41.07	41.07
							p	0.00	0.00	0.00
PS 20:1/22:6	C48 H80 N O10 P	860.5447	0.21±0.15	0.00±0.00	0.56±0.35	0.00±0.00	F	2.54	12.72	2.54
							p	0.15	0.01	0.15
PS 22:6/22:6	C50 H74 N O10 P	878.4978	4.22±0.20	0.00±0.00	0.00±0.00	0.00±0.00	F	1051.70	1051.70	1051.70
							p	0.00	0.00	0.00
C20:5 PS			34.07±1.29	52.70±10.87	61.13±5.07	70.42±4.49	F	35.46	13.64	1.44
							p	0.00	0.01	0.26
C22:6 PS			38.92±2.68	0.00±0.00	23.42±7.48	14.27±3.96	F	0.05	68.42	28.60
							p	0.83	0.00	0.00
C20:4 PS			22.05±0.96	47.30±10.87	11.34±0.75	13.26±0.28	F	44.91	18.86	14.10
							p	0.00	0.00	0.01
Phosphatidylinositols (% from total PI)										
		$[M-H]^-$								
PI 16:0/20:5	C45 H77 O13 P	855.5029	8.93±0.94	3.61±0.54	9.14±3.88±	6.93±0.69	F	2.25	10.18	1.74
							p	0.17	0.01	0.22
PI 16:0/20:4	C45 H79 O13 P	857.5186	4.76±0.89	7.101.58	2.14±0.33	1.69±0.40	F	54.33	3.05	6.55
							p	0.00	0.12	0.03
PI 17:0/20:5	C46 H79 O13 P	869.5186	3.15±0.84	0.91±0.33	2.51±0.50	2.11±0.36	F	0.77	17.48	8.52
							p	0.41	0.00	0.02
PI 17:0/20:4	C46 H81 O13 P	871.5342	1.38±0.36	2.72±0.29	0.65±0.07	0.23±0.26	F	110.01	9.09	33.29
							p	0.00	0.02	0.00
PI 18:2/20:5	C47 H77 O13 P	879.5029	0.84±0.06	0.00±0.00	0.31±0.07	0.00±0.00	F	92.08	434.19	92.08
							p	0.00	0.00	0.00
PI 18:1/20:5	C47 H79 O13 P	881.5186	11.79±0.98	11.42±2.99	28.39±0.90	30.91±0.58	F	354.71	1.27	2.29
							p	0.00	0.29	0.17
PI 18:0/20:5	C47 H81 O13 P	883.5342	44.45±3.11	43.64±2.57	33.56±2.00	36.29±0.48	F	48.54	0.54	1.83
							p	0.00	0.48	0.21
PI 18:0/20:4	C47 H83 O13 P	885.5499	19.51±1.46	23.04±3.98	7.77±0.91	8.63±0.27	F	108.53	3.06	1.13
							p	0.00	0.12	0.32
PI 18:0/20:3	C47 H85 O13 P	887.5655	0.38±0.19	1.19±0.43	0.76±0.17	0.00±0.00	F	8.01	0.03	29.76
							p	0.02	0.87	0.00
PI 18:0/20:2	C47 H87 O13 P	889.5812	0.00±0.00	0.00±0.00	0.52±0.16	0.00±0.00	F	31.60	31.60	31.60

							p	0.00	0.00	0.00
PI 19:1/20:5	C48 H81 O13 P	895.5342	0.19±0.09	0.00±0.00	1.33±0.14	1.16±0.09	F	462.16	11.52	0.01
							p	0.00	0.01	0.92
PI 19:0/20:5	C48 H83 O13 P	897.5499	1.19±0.43	1.47±0.24	2.22±1.16	2.39±0.28	F	6.80	0.36	0.02
							p	0.03	0.56	0.89
PI 19:0/20:4	C48 H85 O13 P	899.5655	0.67±0.36	1.33±1.09	0.84±0.18	0.28±0.14	F	1.68	0.02	3.25
							p	0.23	0.89	0.11
PI 20:5/20:5	C49 H75 O13 P	901.4873	0.31±0.05	0.00±0.00	0.14±0.12	0.00±0.00	F	5.30	35.39	5.30
							p	0.05	0.00	0.05
PI 40:8	C49 H79 O13 P	905.5186	0.50±0.05	0.00±0.00	0.00±0.00	0.00±0.00	F	350.95	350.95	350.95
							p	0.00	0.00	0.00
PI 18:1/22:6	C49 H81 O13 P	907.5342	0.87±0.18	0.78±0.37	1.06±0.25	0.91±0.23	F	1.15	0.58	0.03
							p	0.31	0.47	0.86
PI 20:1/20:5	C49 H83 O13 P	909.5499	0.81±0.26	1.77±0.23	6.23±0.93	6.53±0.82	F	186.54	2.85	0.75
							p	0.00	0.13	0.41
PI 20:1/20:4	C49 H85 O13 P	911.5655	0.28±0.18	1.03±0.67	2.48±0.49	1.93±0.73	F	23.08	0.09	4.04
							p	0.00	0.77	0.08
C20:5 PI			71.67±2.66	62.82±6.52	83.83±1.76	86.33±0.87	F	71.47	2.26	7.24
							p	0.00	0.17	0.03
C20:4 PI			26.59±2.28	35.21±6.10	13.87±1.31	12.75±1.08	F	81.88	3.73	6.28
								0.00	0.09	0.04

Data were presented as mean ± SD, n = 4

[1] *sn*-1(2) acyl/ *sn*-2(1) acyl

[2] *sn*-1 alkyl(alkenyl)/ *sn*-2 acyl

Table S5. Molecular species composition of sphingomyelins, phosphatidic acids and lyso- glycerophosphoethanolamine in in muscle and hepatopancreas of crabs *Eriocheir japonica* and *Paralithodes camtschaticus*

Molecular species	Formula	Detected m/z	*Eriocheir japonica* Muscle	Hepatopancreas	*Paralithodes camtschaticus* Muscle	Hepatopancreas
Sphingomyelins (% from total SM)						
		$[M+H]^+$				

C37 H75 N2 O6 P		675.5436	25.08±5.79	39.74±8.44	33.84±2.70	26.30±4.39
C38 H77 N2 O6 P		689.5607	11.34±2.17	2.05±0.54	5.56±1.18	0.69±0.36
C39 H79 N2 O6 P		703.5749	26.31±5.43	29.14±3.08	14.00±2.53	13.30±1.89
C40 H81 N2 O6 P		717.5909	7.37±0.88	0.00±0.00	1.13±0.51	0.00±0.00
C41 H81 N2 O6 P		729.5904	0.00±0.00	7.38±2.79	27.49±1.61	38.49±1.91
C41 H83 N2 O6 P		731.6072	19.76±2.83	21.70±6.80	4.04±1.00	1.83±0.71
C42 H85 N2 O6 P		745.6234	3.90±0.30	0.00±0.00	0.28±0.21	0.00±0.00
C43 H85 N2 O6 P		757.6218	0.00±0.00	0.00±0.00	8.70±2.72	17.65±4.00
C43 H87 N2 O6 P		759.6349	6.24±0.54	0.00±0.00	1.11±0.48	0.00±0.00
C45 H89 N2 O6 P		785.6494	0.00±0.00	0.00±0.00	3.85±0.98	1.75±0.62
Phosphatidic acids (% from total PA)						
		$[M-H]^-$				
PA 17:0/16:1; 17:0/16:1[1]	C36 H69 O8 P	659.4661	0.27±0.11	0.00	3.34±1.08	0.00±0.00
PA 34:1	C37 H71 O8 P	673.4792	4.10±1.59	0.00	0.67±0.19	0.00±0.00
PA 17:0/18:1; 16:0/19:1	C38 H73 O8 P	687.4964	2.77±0.56	0.00	20.07±6.97	61.86±12.04
PA 18:1/18:1	C39 H73 O8 P	699.5017	3.13±0.73	100.00±0.00	0.09±0.15	0.00±0.00
PA 17:0/20:5	C40 H69 O8 P	707.4635	0.99±0.32	0.00	7.84±1.70	9.75±1.68
PA 18:1/18:1	C40 H75 O8 P	713.5097	0.88±0.39	0.00	2.43±0.84	0.00±0.00
PA 39:7e[2]	C42 H71 O7 P	717.4878	0.00±0.00	0.00	0.47±0.22	0.00±0.00
PA 19:2e/20:4	C42 H73 O7 P	719.5014	8.69±2.05	0.00	1.70±0.70	0.00±0.00
PA 18:1/20:5	C41 H69 O8 P	719.4638	12.47±4.21	0.00	0.13±0.03	0.00±0.00
PA 18:0/20:5	C41 H71 O8 P	721.4781	41.91±9.07	0.00	10.54±2.77	0.00±0.00
PA 19:1/20:5; 18:1/21:5	C42 H71 O8 P	733.4797	4.17±1.64	0.00	26.87±8.44	19.48±5.92
PA 39:5	C42 H73 O8 P	735.495	6.99±1.49	0.00	14.96±4.02	7.57±2.62
PA 19:0/20:4	C42 H75 O8 P	737.5101	2.74±0.68	0.00	0.00±0.00	0.00±0.00
PA 41:7e	C44 H75 O7 P	745.5174	0.00±0.00	0.00	0.89±0.38	0.00±0.00
PA 40:7	C43 H71 O8 P	745.4775	8.55±1.41	0.00	0.73±0.19	0.00±0.00
PA 41:6e	C44 H77 O7 P	747.5321	0.00±0.00	0.00	0.35±0.09	0.00±0.00
PA 20:5/21:5	C44 H67 O8 P	753.45	0.00±0.00	0.00	1.09±0.29	0.00±0.00
PA 19:1/22:6	C44 H73 O8 P	759.4947	1.12±0.56	0.00	4.65±1.03	1.34±0.52
PA 41:6	C44 H75 O8 P	761.5076	1.23±0.24	0.00	3.20±0.80	0.00±0.00
Lyso glycerophosphatidylethanoamines (% from total LPE)						
		$[M-H]^-$				
LPE 16:1e	C21 H44 N O6 P	436.2834	0.00±0.00	6.35±1.18	2.26±0.78	0.32±0.18

LPE 18:2e	C23 H46 N O6 P	462.2992	3.88±0.67	0.00±0.00	15.06±5.75	0.74±0.21
LPE 18:1e	C23 H48 N O6 P	464.3132	16.91±4.94	29.56±3.88	7.74±2.44	1.40±0.56
LPE 18:1	C23 H46 N O7 P	478.2921	58.53±7.35	0.00±0.00	7.32±1.81	0.00±0.00
LPE 20:5	C25 H42 N O7 P	498.2604	13.75±2.17	44.62±8.49	29.18±8.49	46.57±3.69
LPE 20:4	C25 H44 N O7 P	500.2762	0.00±0.00	19.47±3.59	5.56±1.84	7.95±1.00
LPE 22:6	C27 H44 N O7 P	524.277	6.93±1.74	0.00±0.00	27.42±4.83	35.91±1.07
LPE 22:5	C27 H46 N O7 P	526.293	0.00±0.00	0.00±0.00	5.47±0.91	7.12±1.57

Data were presented as mean ± SD, *n* = 4

[1] *sn*-1(2) acyl/ *sn*-2(1) acyl

[2] *sn*-1 alkyl(alkenyl)/ *sn*-2 acyl
